# Physically Modified Plant Oils as Alternatives to Palm Fat: Effects on Physical and Flavour Properties of Chocolate Fillings

**DOI:** 10.3390/foods14071179

**Published:** 2025-03-28

**Authors:** Tamara Schmid, Elodie Gillich, Amandine André, Mathias Kinner, Irene Chetschik, Nadina Müller

**Affiliations:** Institute of Food and Beverage Innovation, Zurich University of Applied Sciences, Einsiedlerstrasse 35, 8820 Wädenswil, Switzerland; tamaraschmid@bluewin.ch (T.S.); elodie.gillich@zhaw.ch (E.G.); amandine.andre@zhaw.ch (A.A.); irene.chetschik@zhaw.ch (I.C.)

**Keywords:** Pickering emulsion, crystallisation, viscosity, chocolate filling, chocolate flavour

## Abstract

Palm and palm kernel oils are extensively utilised in food processing due to their unique properties, such as their semi-solid consistency at room temperature. However, growing environmental and social concerns regarding palm oil production have prompted the industry to seek sustainable alternatives to tropical or hydrogenated fats. This project investigated the use of plant oils and their emulsified and crystallised forms as potential substitutes for palm fat in light and dark chocolate fillings, with an emphasis on single-origin ingredients to align with clean-label trends. The emulsions were assessed for viscosity, firmness, colour, and key flavour and aroma profiles. Results demonstrated that palm fat alternatives performed effectively in dark chocolate fillings, with non-emulsified recipes achieving firmness comparable to palm fat. In contrast, light chocolate fillings exhibited reduced firmness at higher inclusion rates of alternatives, and emulsified products were prone to flocculation. Notably, pure oil formulations delivered promising outcomes at lower inclusion rates, as the firmness could be raised by 22.0%, likely due to vegetable oil and cocoa butter interactions influencing crystal morphology. Substituting palm oil with sunflower oil, either crystallised or emulsified, did not compromise the overall flavour. Future investigations should determine the maximum feasible level of palm fat substitution and investigate the potential of adding higher amounts of waxes and emulsifiers to enhance crystal formation and firmness.

## 1. Introduction

Palm and palm kernel oils are extensively utilised in food processing due to their unique properties, such as their semi-solid consistency at room temperature and favourable melting behaviour [1]. These oils have widespread application in products such as baked goods, fried foods, and chocolate fillings [2]. In chocolate fillings, palm kernel oil from the fruit kernel is often preferred over palm oil, which is derived from the fruit’s pulp, because of its narrower and lower melting range [3]. Despite the high oil yield per land area, there is concern about additional tropical deforestation as well as peatland draining and burning to cover the additional demand for palm oil [4]. The resulting increasing environmental and social concerns related to the production and sourcing of palm oil have prompted efforts within the industry to seek sustainable alternatives to tropical or hydrogenated fats.

Efforts to replace palm fat in food products have involved several approaches, including the development of emulsions and particle-stabilised emulsions [5,6,7], crystallised oils and emulsions [8,9,10,11] as well as oleogels and hydrogels [12,13,14,15]. While a wide variety of surface-active particles are available for food applications [16,17], the range of waxes used for crystallisation is limited, with candelilla, carnauba, rice bran and sunflower wax being the most commonly used types [18]. Toro-Vazquez et al. [19] demonstrated that wax concentrations between 1% and 4% can improve crystallisation behaviour, whereas Liu et al. [20] only observed beneficial effects at concentrations exceeding 6%. Husmann et al. [5] explored the use of native waxes derived from oil extraction press cakes for targeted crystallisation of oils and their emulsions, and found an increased viscosity and enhanced long-term stability at a wax concentration of 0.5%, but suggested that higher concentrations of 1% could yield even better results.

Various studies have focused on evaluating palm fat alternatives in chocolate-related applications. For instance, Oba and Yildirim [21] tested oleogels made from pistachio, wheat, fig seed, flaxseed, and pumpkin seed oils in chocolate spreads. They found that pistachio-based oleogels achieved promising results with 75–100% palm fat replacement, while the replacement ratio for wheat, fig seed, and flaxseed oil-based oleogels was limited to 25% due to insufficient firmness. Similarly, shellac oleogels were successfully employed as partial palm fat replacements in chocolate, particularly to reduce fat migration upon the addition of oils [22]. Recent studies have also explored hydrogels made from proteins or polysaccharides, as these are often better suited for food applications than oleogels, although they may increase the risk of sugar bloom [15].

The use of water-in-cocoa butter emulsions has also been studied as a method for reducing calorie content in chocolate while avoiding the grittiness caused by direct water incorporation ([23], 2010). Prosapio and Norton [24] developed a calorie-reduced chocolate containing 40% less fat through the use of water-in-cocoa butter emulsions, demonstrating that the amount of sugar dissolved in water significantly affects droplet size. Norton and Fryer [7] further showed that emulsifier dosages of up to 5% improve droplet size and emulsion stability. Di Bari et al. [25] investigated the influence of processing conditions on the microstructure of water-in-cocoa butter emulsions, finding that typical margarine-producing equipment, such as scraped-surface heat exchangers, could be used to temper cocoa butter during emulsification. They also noted that higher rotor speeds in such equipment yield smaller droplet diameters.

The unique aroma of cocoa arises from the biochemical and thermal conversion of precursors within the cocoa beans during fermentation and across the entire cocoa value chain. In the chocolate industry, substitutes for cocoa butter—such as palm oil-based products—are often used to achieve specific melting properties without compromising this distinctive aroma. These substitutes are typically neutral in flavour, ensuring they do not interfere with the overall cocoa aroma in the final product [26]. However, the effects of other plant oils, such as sunflower or rapeseed, and their particle-stabilised forms, as cocoa butter substitutes in chocolate fillings, have not yet been explored.

This study investigates the use of plant oils and their particle-stabilised emulsions and crystallised forms as alternatives to palm fat in light and dark chocolate fillings, which are products of high importance in the confectionery sector. By limiting raw material use to components derived exclusively from oil plants, this research introduces a novel single-origin ingredient strategy aimed at promoting clean-label products. Moreover, as this study focuses on rapeseed and sunflower vegetable oils, this approach could lead to a palm oil alternative which is locally produced and therefore a more sustainable product. The emulsions were characterised in terms of viscosity during heating and cooling, firmness after cooling, colour, and key tastants and aroma compounds.

## 2. Materials and Methods

### 2.1. Raw Materials

#### 2.1.1. Palm Fat and Ingredients of Plant Oil Emulsions

The palm fat alternatives were prepared using either rapeseed oil (HOLL Rapsöl, Florin AG, Muttenz, Switzerland), finely milled rapeseed press cake (Rapspresskuchen, Florin AG, Muttenz, Switzerland), and rapeseed wax (Raps-Wachspastillen, Graine Creative, Preuilly-sur-claise, France), or sunflower seed oil (Sonnenblumenöl Knospe, Nutriswiss AG, Lyss, Switzerland), finely milled sunflower seed press cake (Sonnenblumenpresskuchen, Nutriswiss AG, Lyss, Switzerland), and sunflower seed wax (Sonnenblumenwachs, Armonia GmbH, Sargans, Switzerland). The palm fat (Eulip, Patiswiss AG, Gunzgen, Switzerland) served as the reference raw material throughout the study.

#### 2.1.2. Chocolate Fillings

For chocolate fillings, the raw materials included rolling stock (Walzgut, Patiswiss AG, Gunzgen, Switzerland), palm fat (Eulip, Patiswiss AG, Gunzgen, Switzerland), dark chocolate mass (CV Dunkel, Patiswiss AG, Gunzgen, Switzerland), light chocolate mass (CV Milch, Patiswiss AG, Gunzgen, Switzerland), and liquid soy lecithin (Sojalecithin rein flüssig, Patiswiss AG, Gunzgen, Switzerland).

#### 2.1.3. Chemicals for Sample Preparation, Extraction, and Quantitation of Polyphenols and Alkaloids

Acetone (LC-MS grade) and water (LC-MS grade) were obtained from Carl Roth AG (Arlesheim, Switzerland). Acetonitrile and methanol (LC-MS grade) were supplied by Honeywell Deutschland Holding GmbH (Offenbach, Germany). Formic acid, theobromine, and caffeine were purchased from Sigma-Aldrich (Merck AG, Zug, Switzerland). n-Hexane was provided by VWR International GmbH (Dietikon, Switzerland). Catechin, procyanidin B2, and cinnamtannin A2 were sourced from Phytolab GmbH & Co. KG (Vestenbergsgreuth, Germany), while epicatechin and procyanidin C1 were purchased from Extrasynthese (Genay, France).

#### 2.1.4. Chemicals for Sample Preparation, Extraction, and Quantitation of Selected Aroma Compounds

Diethylether (99.5% Rotipuran) and anhydrous sodium sulfate (≥99%, p.a.) were supplied by Carl Roth AG (Arlesheim, Switzerland).

The stable isotopically substituted odorants were sourced from AromaLAB GmbH (Martinsried, Germany), including 2-(2H3)methylbutanal, 3-methyl(3,5-2H2)butanal, ethyl 2-(2H3)methylbutanoate, ethyl 3-(2H3)methyl(2,2,3,4,4,4-2H6)butanoate, (2H6)dimethyl trisulfide, 2-(2H5)ethyl-3,6-dimethylpyrazine, 2-(2H3)methyl-3,5-dimethylpyrazine, 3-(2H3)methyl-(2,2,3,4,4,4-2H6)butanoic acid, ethyl 2-(2H5)phenylacetate, 3-methylbutyl-(13C2)acetate, 2-(2H5)phenylethanol, 2-(2H3)methoxyphenol, 4-methyl(2,6-2H2)phenol, 4-hydroxy-2-methyl-5-(13C)methyl(5-13C)furan-3(2H)-one, 3-hydroxy-4-methyl-5-(13C)methyl(5-13C)furan-2(5H)-one, 5-(4,4,5,5-2H2)pentyloxolan-2-one, 3(2H3)methyl-7-methyl-(4,4-2H2)octa-1,6-dien-3-ol, and phenyl(13C2)acetaldehyde. Merck KGaA (Darmstadt, Germany) provided (13C2)acetic acid.

The reference odorants 2-methylbutanal, 3-methylbutanal, ethyl 2-methylbutanoate, ethyl 3-methylbutanoate, dimethyl trisulfide, 2-ethyl-3,(5 or 6)dimethylpyrazine, 2,3,5-trimethylpyrazine, 2-methylbutanoic acid, 3-methylbutanoic acid, ethyl 2-phenylacetate, 3-methylbutyl acetate, 2-phenylethanol, 2-methoxyphenol, 4-methylphenol, 4-hydroxy-2,5-dimethylfuran-3(2H)-one (furaneol), 3-hydroxy-4,5-dimethylfuran-2(5H)-one (sotolon), 5-pentyloxalan-2-one (γ-nonalactone), 3,7-dimethylocta-1,6-dien-3-ol (linalool), phenylacetaldehyde, hexanal, and acetic acid were purchased from Merck KGaA.

### 2.2. Production Methods

#### 2.2.1. Formation of Particle-Stabilised and/or Crystallised Palm Fat Alternatives

**Figure 1:**
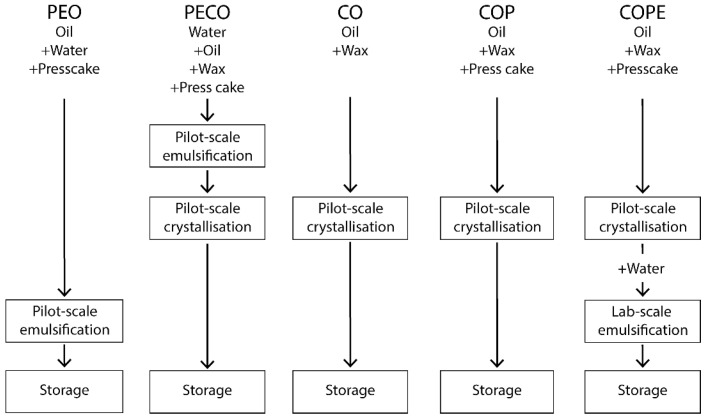
Five different processes for producing palm fat alternatives for use in chocolate fillings: CO stands for ‘Crystallised Oil’, COP for ‘Crystallised Oil with Press cake’, PECO for ‘Particle-stabilised Emulsified Crystallised Oil’, COPE for ‘Crystallised Oil with Particle Emulsification’ and PEO for ‘Particle-stabilised Emulsified Oil’.

Five distinct processes for producing palm fat alternatives were tested as substitutes for palm fat in chocolate fillings. These processes are visualised in Figure 1.

##### Preparation of Suspensions

For the CO and COP processes, 99.5% (*v*/*v*) oil was measured, and 0.5% (*w*/*v*) wax was added to 1/5th of the oil. The portion containing wax was then heated on a stove to 85 °C and held at that temperature until the wax was completely melted. For the COP process, another 1/5th of the oil was mixed with 5% (*w*/*v*, based on the total amount of the 100% liquid phase) press cake using a mixer (Rotor Lips AG, Uetendorf, Switzerland, GT 800) until it was homogenously dispersed.

For PEO, 1/5th of the oil was combined with 5% *(w*/*v)* press cake, calculated based on the total water-oil emulsion, and mixed in the same mixer (Rotor Lips AG, Uetendorf, Switzerland, GT 800) until it was homogenously dispersed. The two or three components were then combined (CO: oil–wax and the remainder of the oil; COP: oil–wax, oil press cake, and the remainder of the oil; PEO: oil press cake and the remainder of the oil) in a bucket and further homogenised using a polytron (Chemcol, Ytron Process Technology GmbH & Co. KG, Bad Endorf, Germany) at 4280 s^−1^ for 5 min. The resulting suspension (oil–wax, oil–wax press cake, or oil press cake) was directly used for crystallisation or emulsification. To ensure consistency, the suspensions were only utilised when their temperature was between 20 °C and 25 °C; otherwise, they were cooled further directly in the crystallisation or emulsification pilot plant tank.

##### Lab-Scale Emulsification

The 214.3 g oil–wax press cake suspension was emulsified with 35.7 g of tap water in a beaker. To prepare the mixture, the suspension and water were placed into a beaker and allowed to rest for 60 s to allow the water to settle at the bottom. The two phases were then emulsified at a shear rate of 9529 s-1 using a polytron (PT 2500 E, Configuration: PT-DA 12/2EC-E157, Kinematica AG, Malters, Switzerland) for 4 min. The emulsification process began in the oil phase, after which the emulsifying device was gradually lowered into the water phase to form a W/O emulsion.

##### Pilot-Scale Emulsification

The suspension (oil press cake for PEO or oil–wax press cake for PECO) was emulsified continuously with 15.0% *(v*/*v)* tap water using a rotor-stator pilot plant (Megatron MT-FM 50, configuration: MTG 45 FFV/6 So, Kinematica AG, Malters, Switzerland) at a shear rate of 95,086 s^−1^. Following emulsification, the PEO emulsion was pumped into baskets, while the PECO emulsion was pumped directly into the scraped surface freezer (SWT-20-RH, Kinematica AG, Malters, Switzerland) for crystallisation (see section ‘Pilot-scale crystallisation’).

##### Pilot-Scale Crystallisation

The suspension or emulsion, at room temperature, was pumped continuously into the freezing area of the scraped surface freezer, where scrapers removed the frozen product from the wall. The freezer was cooled using an external cooling device (Unistat 430, Peter Huber Kältemaschinenbau SE, Offenburg, Germany) with ethanol as the cooling agent, maintaining a cooling temperature of −30 °C. The flow rate of the suspension or emulsion was set to 30 L/h, resulting in a holding time of 36 s. The scraper speed was set to 100 rpm, with a radius of 0.056 m and a gap of 0.002 m between the scraper and the jacket surface, yielding an estimated shear rate of 147 s^−1^. The outlet temperature of the frozen product ranged between −5 °C and −10 °C.

#### 2.2.2. Preparation of Chocolate Filling

##### Chocolate Filling Preparation

Lab-Scale Preparation

Each trial used a total mass of 1 kg. The exact recipes are listed in Table 1. For both the dark and light chocolate fillings, the rolling stock was mixed with one-third of the palm fat or the palm fat alternative in a blender (Thermomix, Vorwerk, Wuppertal, Germany) for 1 min at level 5 of 10. The remaining palm fat or palm fat alternative, along with the dark or light chocolate mass and liquid soy lecithin was then added and mixed in the Thermomix for 5 min at level 5.

The chocolate filling was tempered manually on a marble plate. First, one-third of the chocolate filling was poured onto the plate, allowed to crystallise, and then incorporated back into the remaining two-thirds of the filling. After mixing, another one-third of the filling was poured onto the marble plate, crystallised, and reincorporated into the rest. Following a final mix, the filling was poured into a container to create a 2 cm thick layer. The container was stored at 4 °C for 1 h to complete crystallisation and subsequently kept at 18 °C until analysis.

Pilot-Scale Preparation

At the pilot scale, only the dark chocolate filling was tested. A total of 10.08 kg of rolling stock was mixed with one-third of 0.84 kg of palm fat or one third of the 0.84 kg of a palm fat alternative in a cutter (Stephan Universalmaschine, A. Stephan und Söhne GmbH & Co, Hameln, Germany) for 5 min at the highest setting. The remaining palm fat or alternative, along with 9.06 kg of dark chocolate mass and 0.02 kg of liquid soy lecithin, was then added and mixed for an additional 12 min at the highest setting. The warm chocolate filling was tempered using a tempering machine (jTop EX, Selmi Group, Pollenzo, Italy) to cool it to 24 °C or manually tempered as described in the lab-scale process.

### 2.3. Analysis

All lab-scale trials were repeated twice on different days, and texture and colour measurements were performed three times per trial (n = 6). The pilot-scale trial with machine tempering was done in continuous mode and two samples were taken with a time interval of thirty minutes between sampling, while manual tempering was done twice. For each pilot trial, texture measurements were repeated three times for each sample, resulting in n = 6 for both methods. Viscosity measurements were repeated three times per sample, with 15 points recorded each time (n = 45). All measurements were conducted at least two days after production to ensure full hardening of the chocolate fillings.

#### 2.3.1. Texture Analysis

The firmness of the chocolate filling was measured using a texture analyser (Texture Analyser TA-XT plus, Stable Micro Systems, Surrey, UK) equipped with a cylindrical punch (5 mm diameter). The test was conducted at a speed of 2 mm/s, measuring the force [N] required to punch the chocolate filling to a depth of 12 mm.

#### 2.3.2. Colour Analysis

Colour was measured with a Chromameter (Chroma meter CR-410, Konica Minolta, Tokyo, Japan). The measurement was done using the L*a*b colour scheme and then for visualisation, it was entered into the ‘Color Hexa’ colour generator (https://www.colorhexa.com/ (accessed on 27 September 2024)). ∆*E* as a quantitative measure of visually detectable differences was computed using the Formula (1):

(1) $$∆E=\sqrt{\left(∆L^{{*}^{2}}+∆a^{{*}^{2}}+∆b^{{*}^{2}}\right)}$$

where L* is the lightness, a* the red/green value, b* the blue/yellow value [27] where differences in perceivable colour can be classified as very distinct (∆E > 3), distinct (1.5 < ∆E < 3) and small difference (∆E > 1.5) [28,29].

#### 2.3.3. Viscosity Analysis

To measure viscosity, samples were heated to 45 °C in a drying oven (Drying and Heating Oven, Binder GmbH, Tuttlingen, Germany) for at least 1 h. Then, 19 mL of chocolate filling was transferred to the sample cup of a rheometer (MCR 702, Anton Paar AG, Graz, Austria) equipped with a coaxial cylindrical measuring system (CC27-SN71788, 64353, Anton Paar AG, Graz, Austria).

Viscosity was measured at a shear rate of 5 s^−1^ across a temperature range from 45 °C to 25 °C measuring 15 points each at 45 °C, 40 °C, 35 °C, 30 °C and 25 °C. Each temperature was held for 30 s, and the samples were cooled at a rate of 5 °C per minute.

#### 2.3.4. Quantitation of Key Tastants and Aroma Compounds in the Chocolate Fillings

Quantitation of cocoa key tastants:

The two alkaloids theobromine and caffeine, as well as the polyphenols catechin, epicatechin, procyanidin B2, procyanidin C1 and cinnamtannin A2 were quantified in the chocolate fillings, in triplicate, using HPLC-UV-MS according to Streule et al. [30]. Pure substances were used for the determination of retention times and for the preparation of calibration solutions (linear ranges: 1–100 μg/mL for catechin, epicatechin and cinnamtannin A2; 1–25 μg/mL for procyanidin B2 and C1; 1–600 μg/mL for theobromine; 1–300 μg/mL for caffeine). Results are expressed as mg/kg of fat free dry matter (ffdm).

Quantitation of cocoa key aroma compounds:

The quantitation of key aroma compounds in the chocolate fillings was conducted following the sample preparation and extraction procedure outlined by Ullrich et al. (2022) [31]. Prior to quantification, extracts from two samples (a reference sample with palm oil and a sunflower-based COPE sample) were prepared and screened with Gas Chromatography-Olfactometry (GC-O) [31] to select the analytes to be quantified (Supplementary Material, Table S1). Quantitation of the selected key aroma compounds was then carried out using GC-MS and GC-GC-MS, following the methodology described by Streule and André [30]. The odorant concentration in the sample was calculated by means of the peak areas of the analyte and the standard, the amount of the added standard, and the sample weight by employing calibration line equations as described by Streule and André [30], and the results were expressed as μg/kg of sample (average of triplicate sample extraction and analysis).

### 2.4. Experimental Design

The study began by testing the application of palm fat alternatives in dark chocolate fillings. Dark chocolate fillings containing sunflower seed or rapeseed-based palm fat alternatives, produced using various emulsification and crystallisation setups, were prepared at lab scale and compared for colour and firmness.

Next, sunflower seed-based crystallised emulsions with and without press cake particles were prepared at the pilot scale. These emulsions were applied in dark chocolate fillings, and the resulting products were analysed for colour, viscosity, texture, and molecular flavour properties.

Finally, crystallised emulsions derived from sunflower seed and rapeseed oils, both with and without press cake particles, were used to produce light chocolate fillings in lab-scale experiments. The colour and firmness of these fillings were compared with palm fat reference samples. For sunflower seed-based recipes, the effect of varying dosages of sunflower seed wax was also tested, with the products evaluated based on firmness and colour.

The results of each unifactorial experimental design were analysed using a Kruskal-Wallis test (α = 0.05) to assess equal group means for the independent variables. Pairwise post-hoc analysis was conducted using an unpaired Wilcoxon test (α = 0.05), and significant differences were indicated with different letters in a compact letter display (e.g., groups marked as a and b are significantly different, whereas a and ab are not).

For the calculations and visualisations, the following software tools and packages were employed: R (version 4.2.1) and RStudio (version 2023.09.0) with the Hmisc (version 5.1-2), spatstat (version 3.0-8), multcompView (version 0.1-10), and ggplot2 (version 3.5.0) packages. For analyses of the key tastants and aroma compounds, XLStat Premium (version 2023.1.5) was used, applying ANOVA with Tukey’s HSD post-hoc tests (α = 0.05).

## 3. Results and Discussion

It was possible to produce high-quality chocolate fillings using all palm fat alternatives, despite the water introduced through the emulsified varieties. Raoufi et al. [32] tested the effect of water and PGPR on compound chocolate after pre-emulsifying water in oil and found that a water addition exceeding 3% disrupted the flow properties of chocolate. Research using water-cocoa butter emulsions to reduce the total fat content in chocolate [33] showed that chocolate with emulsions containing 20% water became too viscous and crumbly [33]. More recent work by Prosapio and Norton [24] demonstrated that, by adjusting the recipe to include sugar in the water phase and modifying the stirring rate, chocolate with properties close to conventional chocolate could be achieved, even with water-cocoa butter emulsions containing up to 40% water [24].

**Figure 2:**
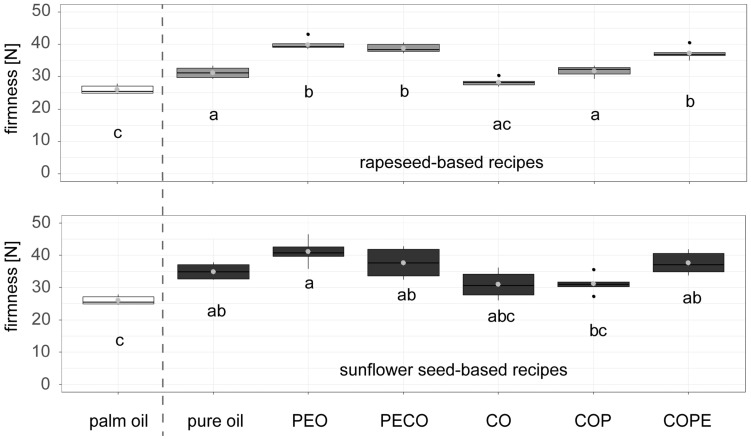
Firmness of dark chocolate filling produced at lab scale from either palm fat or palm fat alternatives based on sunflower seed (oil, press cake, wax)- and rapeseed (oil, press cake, wax)-based recipes: PEO = Particle-stabilised Emulsified Oil, PECO = Particle-stabilised Emulsified Crystallised Oil, CO = Crystallised Oil, COP = Crystallised Oil with press cake, COPE = Crystallised Oil with Particle Emulsification. Firmness was measured with a texture analyser (n = 6). Compact letters (e.g., ab, c) indicate significant differences. White boxplots were used for palm oil based results, grey for rapeseed based and black for sunflower based recipes. Palm oil-based reference samples are shown on the left side of the dashed line, rapeseed- and sunflower seed-based samples on the right side of the dashed line.

Figure 2 shows the firmness of dark chocolate fillings with different palm fat alternatives compared to palm fat. The upper graph presents the palm fat alternatives based on rapeseed recipes, while the lower graph focuses on sunflower seed-based recipes. Both rapeseed and sunflower seed-based alternatives exhibited a consistent trend toward firmer chocolate fillings when prepared with PEO, PECO, and COPE. Notably, there were no significant differences in firmness between the palm fat reference and the CO-based fillings for either raw material. Similarly, sunflower seed-based recipes with COP did not significantly differ from the palm fat reference. Interestingly, all fillings produced with palm fat alternatives have higher firmness than the palm fat reference, despite the palm fat being hard at room temperature and the palm fat alternatives remaining liquid and pourable.

The higher firmness observed in chocolate fillings containing PEO, PECO, and COPE, compared to both the palm fat-based and pure oil-based varieties (CO and COP), might be attributed to the added water phase. Emulsions are known to exhibit higher viscosity than their corresponding oils [34]. The unexpectedly higher firmness of the fillings with pure oil compared to those with palm fat (Figure 2) might result from interactions between the natural chocolate butter in the cocoa mass and the pure sunflower seed or rapeseed oils. Previous research has shown that combining palm fat with 5% soybean or sunflower seed oil alters crystallisation behaviour compared to pure palm fat. Specifically, mixtures of palm fat and sunflower seed oil formed a more organised crystalline network [35].

**Figure 3:**
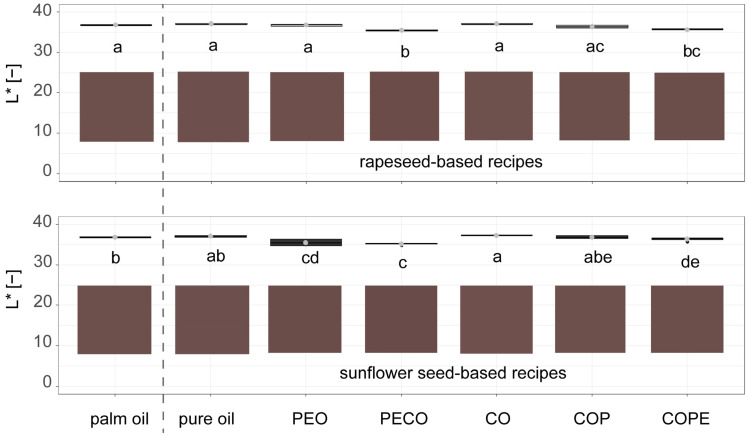
L* values of dark chocolate fillings produced at lab scale with palm fat and alternatives based on sunflower seed (oil, press cake, wax)- or rapeseed (oil, press cake, wax)-based recipes: PEO = Particle-stabilised Emulsified Oil, PECO = Particle-stabilised Emulsified Crystallised Oil, CO = Crystallised Oil, COP = Crystallised Oil with Press cake, COPE = Crystallised Oil with Particle Emulsification (n = 6). The coloured squares are representations of the colours in RGB values converted from the measured L*a*b* values. Compact letters (e.g., ab, c) indicate significant differences. Palm oil-based reference samples are shown on the left side of the dashed line, rapeseed- and sunflower seed-based samples on the right side of the dashed line.

Figure 3 shows the brightness of the different dark chocolate fillings. For the rapeseed-based recipes, only the chocolate fillings produced using the PECO and COPE processes showed significant differences in brightness compared to the palm fat reference. In the lower graph, which displays sunflower seed-based recipes, fillings that were made with PEO and CO, as well as those that were made with PECO and COPE, exhibited significant colour differences compared to the palm fat-based filling.

This indicates that, except for the rapeseed-based recipe with PEO, all chocolate fillings containing a palm fat alternative with water were darker than the recipes without water. This effect might be attributed to the differing light refraction properties of fat and water, as noted in the research by Michels et al. [36]. To answer whether the difference in colour measurement is distinguishable by eye, ∆E  was calculated for each formulation and is shown in Table 2. Based on the definition that ∆E values of 1.5 and more are perceived as a small difference [28], only the samples with PECO-based palm fat alternatives show a small difference in colour, while all other samples do not differ in colour to an extent that is distinguishable by eye.

**Figure 4:**
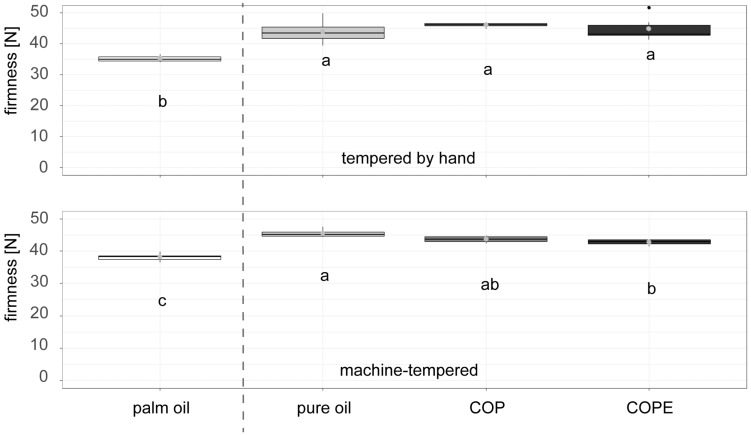
Dark chocolate filling produced at the pilot scale with palm fat and palm fat alternatives based on sunflower seed (oil, press cake, wax)-based recipes: COP = Crystallised Oil with Press cake, COPE = Crystallised Oil with Particle Emulsification. Chocolate fillings in the upper graph were tempered by a machine; the chocolate fillings in the lower graph were tempered by hand (n = 6). Compact letters (e.g., ab, c) indicate significant differences. Palm oil-based reference samples are shown on the left side of the dashed line, sunflower seed-based samples on the right side of the dashed line.

Figure 4 illustrates the firmness of dark chocolate fillings produced at the pilot scale, comparing machine-tempered fillings (upper graph) with those tempered by hand (lower graph) for selected palm fat alternatives. Both tempering methods revealed significantly higher firmness in chocolate fillings containing pure oil, COP, and COPE compared to the palm fat reference.

When tempered by machine, however, a trend emerges: fillings with pure oil exhibited the highest firmness, while fillings with COPE were closest in firmness to the palm fat reference. In contrast, no significant differences were observed among the three palm fat alternatives when tempered by hand. As highlighted in previous research, precise tempering is essential to achieving optimal crystallisation and texture in chocolate ([37,38]). While manual tempering was less precise than machine tempering, the results consistently reflected the same trend of higher firmness for the palm fat alternatives compared to the palm fat reference, aligning with the observations from the machine-tempered samples.

**Figure 5:**
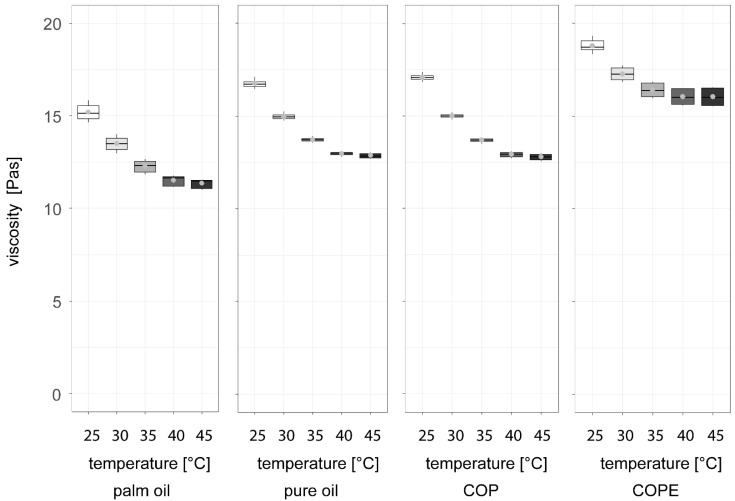
Viscosity measurement with a temperature range from 45 °C to 25 °C of dark chocolate filling produced at the pilot scale with palm fat and alternatives based on sunflower seed (oil, press cake, wax)-based recipes: COP = Crystallised Oil with Press cake, COPE = Crystallised Oil with Particle Emulsification (n = 45).

When melted to 45 °C and subsequently cooled to 25 °C in a rheometer to measure viscosity during cooling, Figure 5 shows that all three chocolate fillings with palm fat alternatives exhibited higher viscosity than the palm fat reference. Notably, while the palm fat alternative filling with COPE was closest in firmness to the palm fat reference (Figure 4, lower graph), its viscosity during cooling from 45 °C to 25 °C was the highest among the alternatives, showing the greatest deviation from the reference. This increased viscosity may result from the combination of water and press cake. According to Schmid et al. [6], sunflower seed press cake contains 27.2 g/100 g fibre [6], and the water-binding capacity of these fibres can contribute to increased viscosity [39]. As the fat crystals were melted during the viscosity measurement, the water-binding capacity of the fibres might have had a stronger impact on the viscosity than the synergistic effects between the cocoa butter and the sunflower seed oil.

Table 3 summarises the quantification of key tastants in the various chocolate fillings. The content of the two cocoa alkaloids, theobromine and caffeine, did not show significant differences between the samples, as all chocolate fillings were made using the same chocolate mass.

For the flavanols catechin, epicatechin, procyanidin B2, procyanidin C1, and cinnamtannin A2, an interesting trend emerged: the chocolate filling produced with palm oil exhibited significantly lower levels of all five quantified flavanols compared to fillings made with sunflower oil, crystallised sunflower oil with press cake (COP), and crystallised sunflower oil with press cake and emulsification (COPE). Since the chocolate mass used in all fillings was identical, these differences suggest that sunflower oil and its press cake contribute significantly higher amounts of these flavanols compared to palm oil.

Supporting this observation, previous studies reported that sunflower press cakes contain total phenolic content ranging from 516 to 810 mg chlorogenic acid equivalent per kg [40]. Additionally, an increase in polyphenol content was noted in bread baked with sunflower press cake [41].

Selected key chocolate aroma compounds were also quantified in the four chocolate fillings to evaluate the impact of sunflower oils and sunflower press cake on their aroma profiles. The compounds analysed included acids (acetic acid, 2-methylbutanoic acid, 3-methylbutanoic acid), fruity esters (ethyl-2-methylbutanoate, ethyl-3-methylbutanoate, ethyl 2-phenylacetate, 3-methylbutylacetate, earthy pyrazines (2,3,5-trimethylpyrazine, 2-ethyl-3,5-dimethylpyrazine), aldehydes (3-methylbutanal, 2-methylbutanal, phenyl acetaldehyde, hexanal), and other key cocoa aroma compounds such as linalool (citrus), 2-phenylethan-1-ol (rose), gamma-nonalactone (coconut), 2-methoxyphenol (smoky) and 4-methylphenol (horse-like), dimethyl trisulfide (cabbage), sotolon (celery, bouillon-like), and furaneol (caramel).

**Figure 6:**
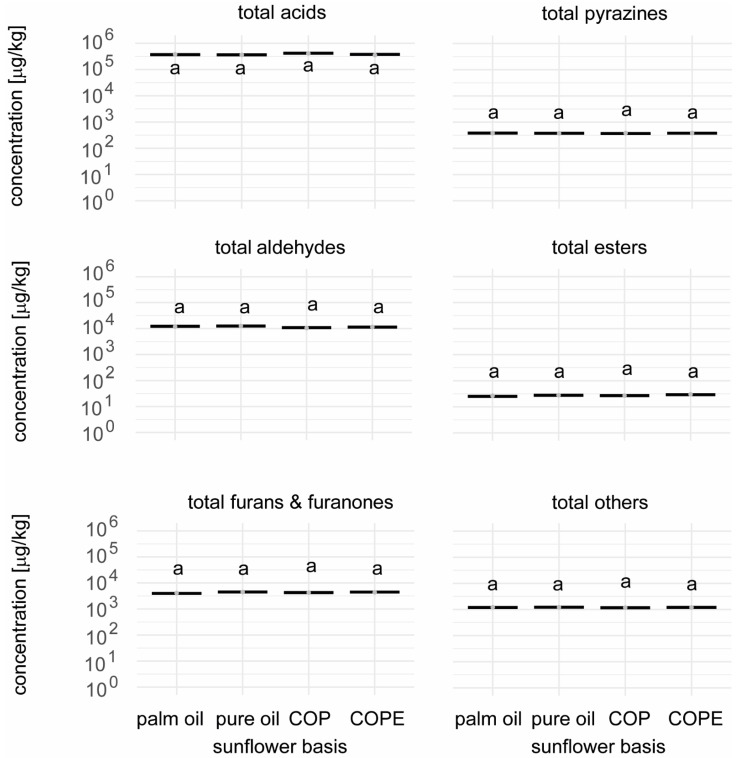
Key aroma compounds quantified in the different chocolate fillings, represented as the sum for each class of compound, expressed as µg/kg sample. ompact letters (e.g., a, b) indicate significant differences (ANOVA, Tukey, α = 0.05).

**Figure 7:**
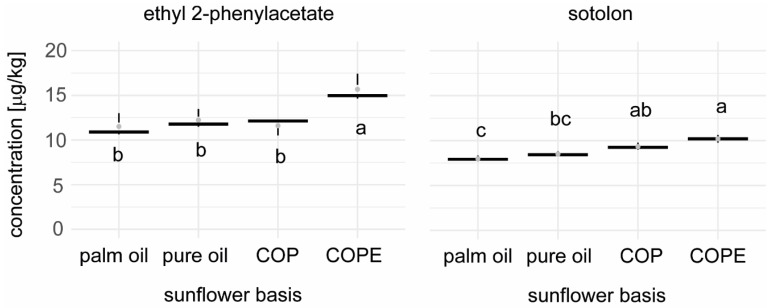
Concentration of ethyl 2-phenylacetate and sotolon in the different chocolate fillings, expressed as µg/kg sample. Compact letters (e.g., ab, c) indicate significant differences (ANOVA, Tukey, α = 0.05).

Among the 21 key aroma compounds quantified, only ethyl 2-phenylacetate (wax-like) and sotolon (celery, bouillon-like) showed significant differences between the samples (Figure 6 and Figure 7; Table S2 in Supplementary Material). Ethyl 2-phenylacetate was only significantly higher in the COPE sample, while sotolon was found in significantly higher amounts in the samples containing sunflower press cake (COP and COPE). When calculating the Dose Over Threshold (DOT) values, ethyl 2-phenylacetate had a DOT value below 1, indicating that this difference had little to no impact on the overall aroma profile of the fillings. Conversely, the DOT value for sotolon exceeded 1, suggesting a potential impact on the aroma profile. During tasting, no differences in aroma were perceived between the fillings despite the presence of soloton. However, these findings would have to be confirmed by a consumer test in a next step. Overall, the quantification of key tastants and key aroma compounds demonstrated that replacing palm oil with sunflower oil, crystallised sunflower oil with press cake, or crystallised sunflower oil with press cake and emulsification had only a minor impact on the flavour quality of the chocolate fillings produced.

Overall, the quantification of key tastants and key aroma compounds demonstrated that replacing palm oil with sunflower oil, crystallised sunflower oil with press cake, or crystallised sunflower oil with press cake and emulsification had no impact on the flavour quality of the chocolate fillings produced.

**Figure 8:**
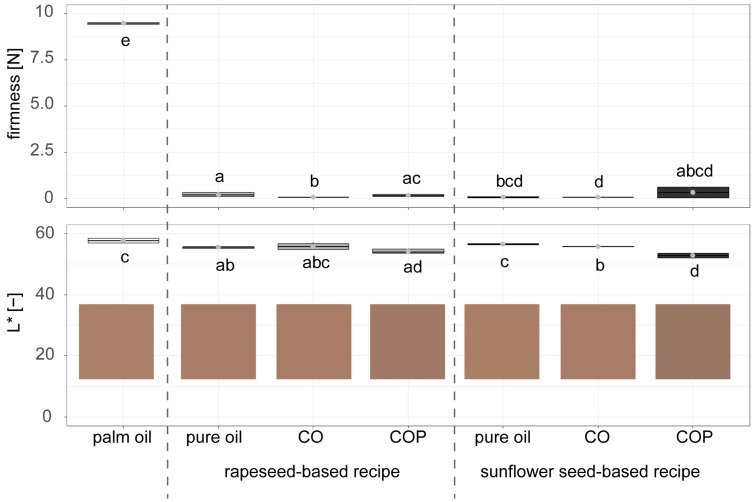
Texture and colour measurement of light chocolate fillings with palm fat an alternatives based on sunflower seed (oil, press cake, wax)- and rapeseed (oil, press cake, wax)-based recipes: CO = Crystallised Oil, COP = Crystallised Oil with Press cake (n = 6). The coloured squares represent the colours in RGB values converted from the measured L*a*b* values. Compact letters (e.g., ab, c) indicate significant differences. The dashed lines mark where palm oil-based reference samples, rapeseed-based and sunflower seed-based results are shown.

**Figure 9:**
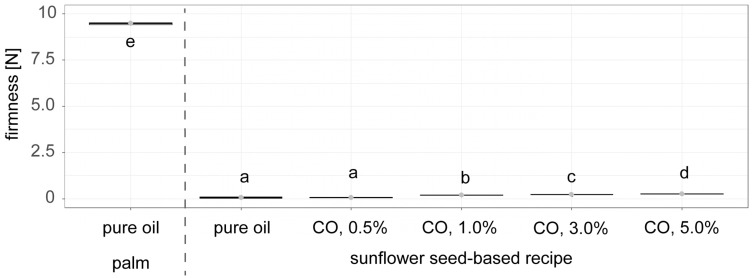
Texture measurement of light chocolate fillings with palm fat and an alternative based on sunflower seed (oil, press cake, wax)-based recipes: CO = Crystallised Oil with different amounts of wax: 0.5%, 1.0%, 3.0%, 5.0% (n = 6). Compact letters (e.g., a, b, c) indicate significant differences. Palm oil-based reference samples are shown on the left side of the dashed line, sunflower seed-based samples on the right side of the dashed line.

Figure 8 shows the analysis of light chocolate fillings made with rapeseed and sunflower seed-based palm fat alternatives compared to the palm fat reference. All trials containing alternatives with water (PEO, PECO, or COPE) coagulated and could not be analysed. Consequently, only results for fillings containing pure oil, CO, and COP are shown in Figure 9.

A significant difference in firmness was observed for both crystallised palm fat alternatives and pure oil compared to the palm fat reference (Figure 8, upper graph). The reference filling with palm fat exhibited a texture more than 40 times firmer than the alternatives. This pronounced effect could be attributed to the high fat content of 20.1% in the light chocolate fillings, where palm fat was replaced with alternatives based on sunflower seed or rapeseed oils. By contrast, the total fat content replaced in the dark chocolate fillings was much lower, at only 4.2% (Figure 2). While the synergistic effect between palm fat and sunflower seed oil described by Chikhoune et al. [35] for a sunflower seed oil content of 5% aligned well with the replacement levels and firmness results in dark chocolate fillings, this effect did not appear to apply to the higher fat content of 20.1% in the light fillings.

The lower graph of Figure 8 shows that both recipes containing COP resulted in significantly darker fillings compared to the palm fat reference. Only the rapeseed-based CO and the sunflower seed-based pure oil fillings displayed no significant differences in colour compared to the reference. The values for ∆E  in Table 4 quantify the colour differences between reference product and light chocolate fillings based on palm fat alternatives to assess whether differences are perceivable by eye. The differences in colour ∆E  of palm fat-based to COP-based palm fat alternatives is larger than 3 and, thus, very distinct [28], while all other palm fat replacements show a distinct difference in colour, defined as 1.5 < ∆E < 3. The darker colour of the fillings containing COP is likely due to the naturally dark colour of the press cake. As noted in the review by Ancuta and Sonia [42], products made with press cakes often exhibit a darker colour [42]. Additionally, an off flavour was identified in the chocolate fillings made with rapeseed-based recipes. Previous research by Szydłowska-Czerniak et al. [43] found a lower overall acceptability and flavour score when rapeseed press cakes were added to biscuits, which aligns with these findings.

Due to the undesirable flavour of the rapeseed-based recipes and their impact on colour, subsequent trials were conducted only with pure oil and CO using sunflower seed-based recipes (Figure 9).

Figure 9 shows the firmness of palm fat alternatives with different amounts of wax content (0.5%, 1.0%, 3.0%, 5.0%) used in light chocolate fillings, compared to pure sunflower seed oil and palm fat. No significant difference in firmness was observed between fillings made with pure oil and those containing 0.5% wax. Although the chocolate fillings with CO containing 1.0%, 3.0%, or 5.0% wax were firmer than those with 0.5% wax, their firmness remained significantly lower than that of the palm fat reference. Furthermore, increasing the wax content from 1.0% to 5.0% resulted in only a slight increase in firmness.

Liu et al. (2019) [20] investigated the effects of candelilla wax and rice wax on crystal morphology, crystallisation kinetics, and product firmness, reporting a notable increase in firmness with higher wax content. Significant differences in firmness were observed for wax additions of 6% and 8% compared to the reference. Microscopic analysis of oil–wax mixtures [5] revealed that sunflower wax forms elongated crystals similar to the rice wax crystals described by Liu et al. (2019) [20]. This suggests that the maximum inclusion level of 5% wax used in this study may have been insufficient, and higher wax levels should be explored in future research.

These findings confirm that for the chosen palm fat alternatives, the amount of palm fat replaced with sunflower seed oil-based alternatives was too high to achieve a firmness comparable to the palm fat reference.

## 4. Conclusions

All tested palm fat replacement approaches performed well in dark chocolate fillings, with non-emulsified recipes tending to match the firmness of palm fat more closely than emulsified plant oil recipes, regardless of whether additional crystallisation was done. At higher inclusion rates in light chocolate fillings, none of the tested palm fat alternatives achieved comparable firmness to the palm fat reference, and all emulsified, water-containing products resulted in flocculation. Interestingly, the use of pure oil produced promising results, likely due to interactions between vegetable oils and cocoa butter influencing crystal morphology, as observed by Chikhoune [35]. The quantification of key tastants and aroma compounds in palm and sunflower-based chocolate fillings confirmed that replacing palm oil with sunflower oil, crystallised sunflower oil with press cake, or crystallised sunflower oil with press cake and emulsification had no impact on the overall flavour properties of the chocolate fillings.

Further research should explore the maximum feasible replacement level of palm fat with vegetable oils, while maintaining the synergistic effects between cocoa butter and vegetable oil. Additionally, the impact of higher wax inclusion levels, possibly combined with emulsifiers to enhance crystal formation, should be investigated to enable a greater replacement of palm fat.

## Figures and Tables

**Table 1:** Recipe to produce the dark as well as the light chocolate filling for chocolate fillings produced in both lab and pilot scale.

Raw Material	Dark Chocolate Filling [%]	Light Chocolate Filling [%]
**Rolling stock**	50.4	63.9
**Palm oil**	4.2	20.1
**Dark chocolate mass**	45.3	0.0
**Light chocolate mass**	0.0	15.9
**Soy lecithin**	0.1	0.1

**Table 2:** ∆E of dark chocolate fillings produced with palm fat alternatives compared to the reference dark chocolate filling produced with palm fat.

∆E	Rapeseed-Based Recipes [–]	Sunflower-Based Recipes [–]
Palm oil—pure oil	0.39	0.54
Palm oil—PEO	0.48	1.29
Palm oil—PECO	1.50	1.61
Palm oil—CO	0.24	0.85
Palm oil—COP	0.44	0.35
Palm oil—COPE	1.22	0.60

**Table 3:** Average content of selected key tastants theobromine, caffeine, catechin, epicatechin, procyanidin B2, procyanidin C1, cinnamtannin A2 in the different chocolate fillings, expressed as mg/kg fat free dry matter (ffdm). Compact letters (e.g., a, b) indicate significant differences (ANOVA, Tukey, α = 0.05).

	Palm Fat	Sunflower Seed-Based Recipe		
	Pure Oil	Pure Oil	COP	COPE
Key tastants	mg/kg fat free dry matter			
Theobromine	2155.71 ± 119.98 ^a^	2181.97 ± 15.1 ^a^	2121.7 ± 119.67 ^a^	2019.42 ± 190.82 ^a^
Caffeine	257.16 ± 15.32 ^a^	259.54 ± 4.17 ^a^	236.31 ± 17.05 ^a^	254.48 ± 13.41 ^a^
Catechin	39.25 ± 5.53 ^b^	65.24 ± 8.69 ^a^	67.73 ± 10.18 ^a^	80.71 ± 4.20 ^a^
Epicatechin	264.99 ± 37.48 ^b^	369.27 ± 48.8 ^a^	374.03 ± 42.19 ^a^	446.15 ± 5.06 ^a^
Procyanidin B2	92.98 ± 13.84 ^b^	160.91 ± 23.74 ^a^	150.37 ± 27.91 ^a^	192.07 ± 9.6 ^a^
Procyanidin C1	76.51 ± 10.53 ^b^	135.38 ± 12.98 ^a^	133.71 ± 26.74 ^a^	176.06 ± 8.31 ^a^
Cinnamtannin A2	10.56 ± 8.68 ^b^	60.3 ± 18.62 ^a^	54.43 ± 20.09 ^a^	87.11 ± 5.04 ^a^

**Table 4:** ∆E of light chocolate fillings produced with the palm fat alternatives compared to the reference chocolate filling produced with palm fat.

∆E	Rapeseed-Based Recipes [−]	Sunflower-Based Recipes [−]
Palm oil—pure oil	2.26	1.96
Palm oil—CO	2.10	2.05
Palm oil—COP	3.55	6.21

## Data Availability

The datasets used and/or analysed during the current study are available from the corresponding author on reasonable request.

## Supplementary Materials

The following supporting information can be downloaded at: https://www.mdpi.com/article/10.3390/foods14071179/s1, Table S1: Results of the GC-O analysis conducted on the palm oil and COPE fillings; Table S2: Quantification of aroma compounds in the 4 different recipes of chocolate fillings. Statistical analysis: one-way ANOVA followed by a Tukey test. Groups with the same letter are not significantly different at α = 0.05.

## Abbreviations

CO	Crystallised Oil
COP	Crystallised Oil with Press cake
PECO	Particle-stabilised Emulsified Crystallised Oil
COPE	Crystallised Oil with Particle Emulsification
PEO	Particle-stabilised Emulsified Oil
