# Supplementary material for: Physically Modified Plant Oils as Alternatives to Palm Fat: Effects on Physical and Flavour Properties of Chocolate Fillings
Source: Foods. 2025 Mar 28;14(7):1179. doi: 10.3390/foods14071179 (PMC11988766; doi:10.3390/foods14071179)
Supplement: Supplementary file 1 [file foods-14-01179-s001.zip › foods-3553737-supplementary.pdf]

Supplementary Material:

Effect of palm fat replacement in chocolate filling with physically modified plant-oils on physical and molecular flavour properties

Tamara Schmid[a], Elodie Gillich[a], Amandine André[a], Mathias Kinner[a], Irene Chetschik[a], Nadina Müller[a*]
[a] Zurich University of Applied Sciences, Institute of Food and Beverage Innovation, Einsiedlerstrasse 35, 8820 Wädenswil, Switzerland

Table S1. Results of the GC-O analysis conducted on the palm oil and COPE fillings.

No.	Compound	Sunflower based - PECO	Palm oil	Odour descriptor	RI-FFAP
1	2- und 3-Methylbutanal	x	x	malty	933
2	2,3-Pentandione	x	x	buttery	1020
3	Hexanal	x	x	herbaceous-green	1057
4	Filbertone	x	x	haselnut	1273
5	1-Octen-3-one	x	x	mushroom	1283
6	Octanal	x	x	citrus	1294
7	2-Acetyl-1-pyrroline	x	x	roasty	1314
8	Dimethyl trisulifde	x	x	cabbage-like	1346
9	2-Propionyl-1-pyrroline	x	x	roasty	1403
10	Acetic acid	x	x	vinegar-like	1421
11	2-Ethyl-3,6-dimethylpyrazine	x	x	earthy	1429
12	Methional	x	x	cooked potato	1437
13	2-Ethyl-3,5-dimethylpyrazine	x	x	earthy	1447
14	Unknown	x	x	earthy	1504
15	(E)-2-Nonenal		x	fatty	1516
16	Linalool	x	x	citrus	1544
17	Isobutyric acid	x	x	pungent-cheesy	1554
18	(E,Z)-2,6-nonadienal	x	x	fatty	1575
19	(Z)-2-Decenal		x	fatty	1597
20	Butyric acid	x	x	pungent-cheesy	1622
21	Phenylacetaldehyde	x	x	flowery	1621
22	2-Methyl-3-(methyldithio)furane	x	x	nutty	1647
23	2- und 3-Methylbutanoic acid	x	x	pungent, rancid	1647
27	Unknown	x	x	fatty	1684
28	(E,E)-2,4-Nonadienal	x	x	fatty	1698
29	(E,E)-2,4-Decadienal	x	x	fatty	1800
30	β-Damascenone	x	x	appel-like	1812
31	Guajacol	x	x	smoky	1849
32	γ-Octalactone	x	x	sweet-coconut-like	1900
33	Unknown	x	x	caramel-like	1911
34	Unknown	x	x	smoky	1945
35	*trans*-4,5-epoxy-(E)-2-decenal	x	x	metallic	2010
36	γ-Nonalactone	x	x	coconut-like	2025
37	Furaneol	x	x	caramel	2039
38	p-Cresol	x	x	horsy-horse stable	2087
39	Unknown	x	x	sweet	2240
40	3-Methylindole	x	x	faecal	2465
41	Vanillin	x	x	vanilla-like	2569

Table S2. Quantification of aroma compounds in the 4 different recipes of chocolate fillings. Statistical analysis: one-way ANOVA followed by a Tukey test. Groups with the same letter are not significantly different at α = 0.05.

	**Palm-based recipe**	**Sunflower-based recipe**		
**Compound/Sample**	**Pure oil**	**Pure oil**	**COP**	**COPE**
	**Concentration µg/kg**			
Acetic acid	374037 ± 31002 a	356948 ± 36850 a	407527 ± 52438 a	351529 ± 46465 a
2-Methylbutanoic acid	1007 ± 0 a	1119 ± 69 a	1001 ± 143 a	916 ± 42 a
3-Methylbutanoic acid	3222 ± 350 a	3146 ± 88 a	2921 ± 161 a	2882 ± 91 a
Total acids	*378266 ± 30921 a*	*361213 ± 36699 a*	*411448 ± 52246 a*	*355326 ± 46423 a*
3-Methylbutanal	8687 ± 533 a	9385 ± 1342 a	7464 ± 835 a	8513 ± 1015 a
2-Methylbutanal	1561 ± 30 a	1647 ± 227 a	1406 ± 44 a	1484 ± 58 a
Phenylacetaldehyde	1549 ± 152 a	1543 ± 104 a	1475 ± 19 a	1437 ± 57 a
Hexanal	138 ± 30 a	131 ± 11 a	137 ± 22 a	127 ± 8 a
Total aldehydes	*11935 ± 563 a*	*12705 ± 1497 a*	*10481 ± 831 a*	*11562 ± 1074 a*
Ethyl-2-methylbutanoate	0.43 ± 0.05 a	0.46 ± 0.01 a	0.46 ± 0.02 a	0.46 ± 0.02 a
Ethyl-3-methylbutanoate	1.27 ± 0.13 a	1.19 ± 0.06 a	1.22 ± 0.14 a	1.16 ± 0.06 a
3-Methylbutylacetate	13.54 ± 1.39 a	14.04 ± 0.36 a	13.66 ± 0.42 a	13.08 ± 0.45 a
Ethyl 2-phenylacetate	11.51 ± 1.3 b	12.24 ± 1.07 b	11.6 ± 0.94 b	15.68 ± 1.52 a
Total esters	*26.76 ± 2.72 a*	*27.93 ± 1.06 a*	*26.94 ± 0.54 a*	*30.37 ± 1.95 a*
2,3,5-Trimethylpyrazine	141 ± 16 a	140 ± 18 a	139 ± 19 a	143 ± 16 a
2-Ethyl-3,5-dimethylpyrazine	241 ± 20 a	236 ± 7 a	244 ± 7 a	243 ± 8 a
Total pyrazines	382 ± 20 a	376 ± 7 a	383 ± 7 a	386 ± 8 a
Furaneol	4101 ± 317 a	4474 ± 124 a	4252 ± 339 a	4351 ± 246 a
Sotolon	7.98 ± 0.32 c	8.49 ± 0.27 b, c	9.33 ± 0.4 a, b	10.2 ± 0.43 a
Total furans, furanones	*4109 ± 20 a*	*4482 ± 7 a*	*4261 ± 7 a*	*4361 ± 8 a*
2-Phenylethan-1-ol	754 ± 38 a	782 ± 44 a	722 ± 38 a	743 ± 10 a
2-Methoxyphenol	13.89 ± 0.67 a	14.17 ± 0.84 a	13.65 ± 0.35 a	14.13 ± 0.66 a
Dimethyl-trisulfide	10.73 ± 0.68 a	11.37 ± 1.59 a	10.68 ± 0.7 a	9.48 ± 0.19 a
Linalool	262 ± 7 a	248 ± 30 a	252 ± 14 a	250 ± 29 a
gamma-Nonalactone	163 ± 2 a	154 ± 19 a	170 ± 6 a	172 ± 25 a
4-Methylphenol	9.74 ± 0.45 a	10.75 ± 1.1 a	9.76 ± 0.26 a	8.6 ± 1.26 a
Total others	*1214 ± 20 a*	*1220 ± 7 a*	*1178 ± 7 a*	*1196 ± 8 a*
